# The Association between Circulating Lipids and Female Infertility Risk: A Univariable and Multivariable Mendelian Randomization Analysis

**DOI:** 10.3390/nu15143130

**Published:** 2023-07-13

**Authors:** Xiaoqi Zhu, Xiang Hong, Jingying Wu, Fanqi Zhao, Wei Wang, Lingling Huang, Jiuming Li, Bei Wang

**Affiliations:** Key Laboratory of Environmental Medicine and Engineering of Ministry of Education, Department of Epidemiology and Health Statistics, School of Public Health, Southeast University, Nanjing 210009, China

**Keywords:** blood lipids, female infertility, mendelian randomization, causal inference

## Abstract

Background: Although observational studies have demonstrated that blood lipids are associated with female infertility, the causality of this association remains unclear. We performed a univariable and multivariable Mendelian randomization (MR) analysis to evaluate the causal relationship between blood lipids and female infertility. Methods: Single-nucleotide polymorphisms associated with lipid traits in univariate analysis were obtained from the Million Veteran Program (MVP) and Global Lipids Genetics Consortium (GLGC), involving up to 215,551 and 188,577 European individuals, respectively. Blood lipids in multivariate analysis were obtained from the latest genome-wide association study meta-analysis with lipid levels in 73 studies encompassing >300,000 participants. Data on female infertility were obtained from the FinnGen Consortium R6 release, which included 6481 samples and 75,450 controls. Subsequently, MR analysis was performed using inverse variance-weighted (IVW), weighted median, weighted-mode, simple-mode and MR-Egger regression to demonstrate the causal relationship between lipids and female infertility. Results: After controlling confounding factors including body mass index and age at menarche, two-sample MR demonstrated that genetically predicted LDL-C and TC were causally associated with the risk of female infertility (When the genetic instruments come from the MVP database, LDL-C and female infertility, IVW OR: 1.13, 95% CI: 1.001–1.269, *p* = 0.047; TC and female infertility, IVW OR: 1.16, 95% CI: 1.018–1.317, *p* = 0.025, and when the genetic instruments came from the GLGC database, LDL-C and female infertility, IVW OR: 1.10, 95% CI: 1.008–1.210, *p* = 0.033; TC and female infertility, IVW OR: 1.14, 95% CI: 1.024–1.258, *p* = 0.015). However, the IVW estimate showed that HDL-C was not significantly associated with the risk of female infertility (when the genetic instruments came from the MVP database, IVW OR: 1.00, 95% CI: 0.887–1.128, *p* = 0.999; when the genetic instruments came from the GLGC database, IVW OR: 1.00, 95% CI: 0.896–1.111, *p* = 0.968). The multivariable MR analysis also provided evidence that LDL-C (OR: 1.12, 95% CI: 1.006–1.243, *p* = 0.042) was significantly associated with the risk of female infertility after considering the correlation of all lipid-related traits. Conclusion: These findings support a causal relationship between increased LDL-cholesterol and increased female infertility risk. Furthermore, the association between lipid-related traits and female infertility risk merits more studies.

## 1. Introduction

Infertility has become a prominent problem in the public health field. Infertility is defined as the inability to achieve a pregnancy after engaging in regular unprotected sexual intercourse for 12 months or longer [1]. An estimated 8 to 12% of couples of reproductive age worldwide face infertility challenges [2]. In China, the rate of infertility in reproductive age is 25%, and nearly half of those who experience infertility have not sought medical attention; this is a prominent clinical problem at present [3,4].

Epidemiological studies have shown that infertility is a fertility disorder caused by multiple etiologies. Evidence from previous studies has confirmed that obesity, alcohol consumption, smoking, education level, and past medical history [3,5,6,7] are associated with female infertility. In addition, infertility may increase the risk of diabetes, cardiovascular disease, and breast cancer [8]. It may also have a chronic impact on women’s mental health, as well as on family cohesion, sexual relationships, and quality of life [9,10].

According to earlier research, abnormal circulating lipid metabolism affects hormonal milieu, steroid synthesis [11], and ovarian and uterine function [12], which in turn has an impact on female reproductive function. A population-based prospective cohort study suggested that serum lipid concentrations may be associated with reduced female fertility and prolonged time-to-pregnancy (TTP) [13]. In addition, a block-randomized, double-blind, placebo-controlled trial reached similar conclusions, with reduced fertility being associated with abnormal female levels of lipids including HDL-C, LDL-C, TC, and TG [14]. Although observational studies have shown a correlation between female lipid levels and infertility, it is unclear whether these correlations are due to the effects of potential biases and confounding factors.

Mendelian randomization (MR) is an experimental design method applied to the field of epidemiology that utilizes genetic variants related to target exposure factors as instrumental variables to assess causality between exposure and outcome [15]. The method follows the Mendelian inheritance law, which states that parental alleles are randomly assigned to offspring. This ensures that the genetic information of individuals is obtained randomly and independently, and is not influenced by the external environment, social behavior, or other factors [16]. A significant benefit of Mendelian randomization is its ability to investigate the enduring effects of exposures on outcomes, such as the impact of lifetime exposure on the risk of disease. Compared to the traditional observational study designs, MR studies are more likely to overcome the influence of confounders and reverse causation [15]. At the same time, although randomized controlled trials (RCTs) are the gold standard for causal inference, it is not feasible in practical situations to use RCTs to study the impact of circulating lipids on female infertility. As a result, it is particularly important to use MR analysis to infer the causal relationship between circulating lipids and female infertility.

In this study, we hypothesize that there may be a possible causal relationship between circulating lipid levels and female infertility. Excluding potentially confounding causal risk factors, we conducted univariable MR to determine whether there was a significant association between lipid traits and female infertility. Meanwhile, given the high dependency between lipid-related traits [17], we further utilized genetic instruments through multivariate MR to compare the independent effect of lipid-related traits on female infertility.

## 2. Materials and Methods

### 2.1. Study Design and Data Sources

**Figure 1:**
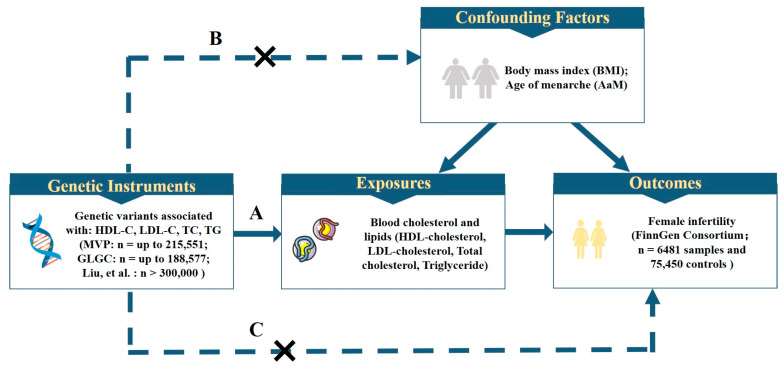
Overview of study design. The design has three key assumptions: (A) the genetic variants should be robustly associated with the lipid–related traits; (B) the genetic variants should not be associated with confounders; (C) the genetic variants influence female infertility only through the risk factors, not via other alternative pathways. HDL–C, high–density lipoprotein cholesterol; LDL–C, low–density lipoprotein cholesterol; MVP, Million Veteran Program; GLGC, Global Lipids Genetics Consortium.

The data information used in the study is shown in Table 1, which includes Ethnicity, Sample Sizes and data sources. The MR study design is displayed in Figure 1, and is based on the following three assumptions: (A) the instrument variables are robustly associated with HDL cholesterol, LDL cholesterol, total cholesterol, and triglycerides; (B) the instrument variables are independent of known confounding factors; and (C) the instrumental variables only affect female infertility through lipid-related traits, and not via other alternative pathways.

Summary genetic data for the univariate analysis, including HDL cholesterol, LDL cholesterol, total cholesterol, and triglycerides, were obtained from the Million Veteran Program (MVP) [18] and the Global Lipids Genetics Consortium (GLGC) [19], which included up to 215,551 European individuals and 188,577 genotyped individuals. MVP is a national research program launched in 2011 to study the impact of genetics, lifestyle, and more on the health of American veterans, and is one of the largest single biological sample banks to date [20]. In the multivariate analysis, summary statistics were selected from the updated GLGC genome-wide association study (GWAS) of 331,368 samples [21] whose sources contain the MVP studies. Some 444 genetic variants were independently associated with at least one of the four lipid-related traits in the GLGC analysis. Genetic variants associated with female infertility were derived from the published GWAS from the FinnGen Consortium (https://r6.finngen.fi/, accessed on 3 February 2023). We used the data from the R6 release, which were diagnosed according to the ICD9 and ICD10. A total of 6481 cases and 68,969 controls were obtained after excluding individuals according to sex.

### 2.2. Genetic Instrument Selection

As genetic variants are commonly used as representative exposure factors in the MR design, the selection of instrumental variables is particularly important for the success of the MR study design. From the MVP lipid study, genetic variants significantly associated with HDL cholesterol, LDL cholesterol, total cholesterol, and triglycerides were selected for genome-wide significance (*p*-values < 5 × 10^−8^). Since body mass index (BMI) [21] and age at menarche (AaM) [21] were associated with female infertility risk, we treated them as confounding factors. Then, we used the PhenoScanner V2 website (http://www.phenoscanner.medschl.cam.ac.uk/, accessed on 7 April 2023) to remove the single-nucleotide polymorphisms (SNPs) that were associated with the two confounding factors (BMI and AaM) or outcome (female infertility), in order to exclude the possibility of genetic pleiotropy. To ensure independence between SNPs, we performed a linkage disequilibrium analysis and removed SNPs with r^2^ > 0.001. Next, the palindromic SNPs with minor allele frequency (MAF) < 1% were removed. In addition, we performed data harmonization to ensure that the effects of SNPs on exposure and outcome corresponded to the same alleles. Detailed information on the genetic instruments used in the univariate MR analysis is shown in Supplementary Tables S1–S8. Considering the genetic and phenotypic interactions between different lipid-related traits, we adjusted for the pleiotropic effects of lipid-related traits including HDL-C, LDL-C, and TG in multivariate MR studies to determine causal associations with female infertility. We performed instrument strength and validity tests to ensure the use of sufficient instrumental variable strength, in which a threshold (F statistics > 10) [15] was used in each multivariable MR experiment. After the screening, a total of 80 single-nucleotide polymorphisms (SNPs) were available in FinnGen (Supplementary Table S9).

### 2.3. Statistical Analysis

We used the inverse variance-weighted method (IVW) as the primary statistical analysis method, with the highest test power under the assumption that there is no horizontal pleiotropy in the instrumental variables [22]. The MR-Egger method [23], weighted median method [24], simple-mode method [25], weighted-mode method [25], and mendelian randomized pleiotropy residual sum and outlier (MR-PRESSO) method [26] were used as secondary analyses. MR-Egger uses a weighted regression approach to estimate the causal effect, and is a statistical method that can be used even when the instrumental variable assumption is invalid. It relies on weaker assumptions, such as the presence of a weak instrument or the absence of measurement error [23]. The assumption of the weighted median method is that a minimum of 50% of genetic instruments are valid instrumental variables that can offer reliable association estimates [24].

In the IVW analysis, we employed the Q statistic to evaluate the existence of heterogeneity among SNPs, and *p*-values < 0.05 were considered to indicate significant heterogeneity [27]. To explore the potential horizontal pleiotropy between the genetic variants and confounders in the MR analysis, we performed an MR-Egger test [28]. In addition, to estimate whether SNPs with outliers and/or pleiotropy would have a potential impact, we performed a leave-one-out analysis using the IVW MR method to detect if the analysis was robust, with stepwise elimination of each SNP [23]. Finally, we used the MR-PRESSO method to further validate the results of the above analysis, which is designed to detect possible outliers and generate estimates after removing outliers, and to perform significant distortion tests of causal estimates before and after correction. In the multivariate MR analysis, IVW is still used as the main statistical analysis method.

We presented the odds ratio (OR) and 95% confidence intervals, as well as all estimates that had two-tailed *p*-values. In the IVW model, *p*-values < 0.05 were considered statistically significant, and the direction of association remained consistent in the other five models (MR-Egger method, weighted median method, simple-mode method, weighted-mode method, MR-PRESSO method). All analyses were performed using the software packages “TwoSample MR” (version 0.5.6, Bristol, UK), “MVMR” (version 0.3, Bristol, UK) and “MR-PRESSO” (version 1.0, New York, NY, USA) in R (version 4.2.1, Vienna, Austria).

## 3. Results

### 3.1. Univariable MR Analysis of Lipid-Related Traits and Female Infertility

**Figure 2:**
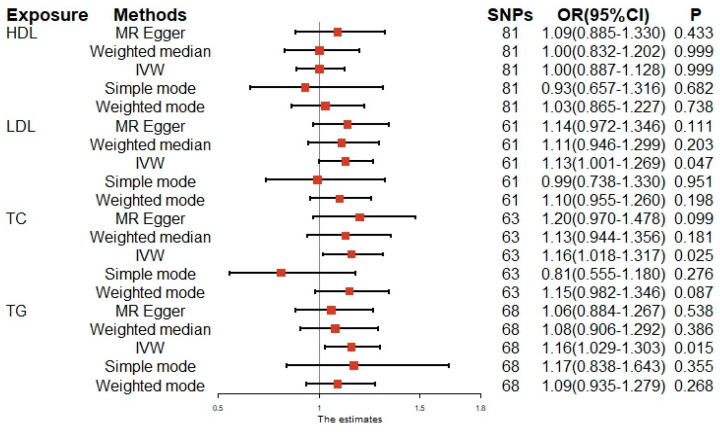
Results of the univariable MR analysis with lipid-related traits from MVP and risk of female infertility. HDL–C, high–density lipoprotein cholesterol; LDL–C, low–density lipoprotein cholesterol; TC, total cholesterol; TG, triglyceride; IVW, inverse variance–weighted; SNP, single–nucleotide polymorphism; OR, odds ratio; CI, confidence interval. MVP, Million Veteran Program.

The relationships of the four lipid-related traits (including HDL-C, LDL-C, TC, and TG) with female infertility in the MVP and FinnGen consortia are shown in Figure 2 and Supplementary Table S10. In the IVW MR analysis, LDL-C (OR: 1.13, 95% CI: 1.001–1.269, *p* = 0.047), TC (OR: 1.16, 95% CI: 1.018–1.317, *p* = 0.025), and TG (OR: 1.16, 95% CI: 1.029–1.303, *p* = 0.015) were associated with an increased risk of female infertility. With a one-unit increase in LDL-C(mmol/L), TC (mmol/L), and TG (mmol/L), the risk of infertility in women increased by 13%, 16%, and 16%, respectively. However, there was no indication that HDL-C (OR: 1.00, 95% CI: 0.887–1.128, *p* = 0.999) was significantly associated with female infertility risk. At the same time, the MR-PRESSO analysis yielded consistent results (Supplementary Table S11). The IVW analysis demonstrated no potential heterogeneity (Supplementary Table S12). Furthermore, the MR-Egger intercepts had *p*-values > 0.1, indicating no directional pleiotropy for genetic variation (Supplementary Table S13). The analysis results of the scatter plots, forest plots, and funnel plots are shown in Supplementary Figure S1A–D, Supplementary Figure S2A–D, and Supplementary Figure S3A–D, respectively. All of the figures are in agreement with the aforementioned analysis results. The leave-one-out analysis indicates that the association between lipid-related traits and female infertility was largely not driven by any single SNP between HDL-C, LDL-C, TC, TG, and female infertility (Supplementary Figure S4A–D).

**Figure 3:**
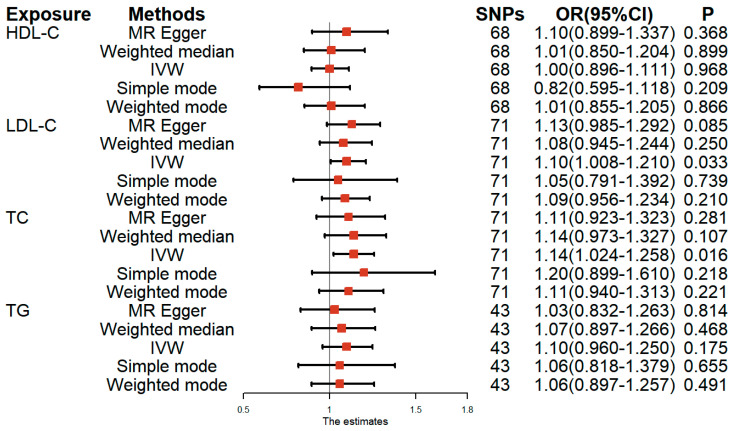
Results of the univariable MR analysis with lipid-related traits from GLGC and risk of female infertility as the outcome. HDL–C, high–density lipoprotein cholesterol; LDL–C, low–density lipoprotein cholesterol; TC, total cholesterol; TG, triglyceride; IVW, inverse variance–weighted; SNP, single–nucleotide polymorphism; OR, odds ratio; CI, confidence interval. GLGC, Global Lipids Genetics Consortium.

To verify whether there was heterogeneity between the MVP and other datasets in terms of the causal relationship with female infertility, we also used four lipid GWASs from GLGC to test the relationship between lipid-related traits and female infertility. Genetically predicted HDL-C (OR: 1.00, 95% CI: 0.896–1.111, *p* = 0.968) and TG (OR: 1.10, 95% CI: 0.960–1.250, *p* = 0.175) were not significantly associated with female infertility risk, as supported by the IVW analysis. However, LDL-C (OR: 1.10, 95% CI: 1.008–1.210, *p* = 0.033) and TC (OR: 1.14, 95% CI: 1.024–1.258, *p* = 0.015) were still causally associated with female infertility risk. Therefore, apart from the different association results of TG, the other results are consistent with the results of MVP (Figure 3 and Supplementary Table S10), as well as the MR-PRESSO analysis (Supplementary Table S11). In addition, neither potential heterogeneity nor directional pleiotropy for genetic variation was found in the analysis using the GLGC dataset (Supplementary Tables S12 and S13). The analysis results of the scatter plots, forest plots, funnel plots, and leave-one-out plots are shown in Supplementary Figures S5–S8.

### 3.2. Multivariable MR Analysis of Lipid-Related Traits and Female Infertility

**Figure 4:**
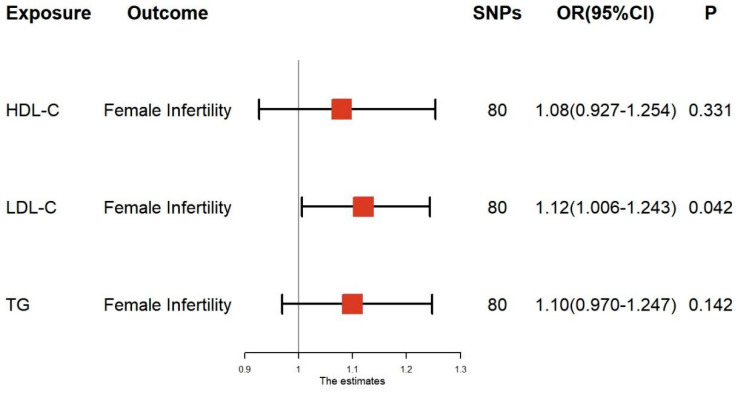
Associations of lipid traits in multivariable MR using the inverse variance–-weighted method. HDL–C, high–density lipoprotein cholesterol; LDL–C, low–density lipoprotein cholesterol; TG, triglyceride; SNP, single–nucleotide polymorphism; OR, odds ratio; CI, confidence interval.

The results of the multivariate MR analysis are shown in Figure 4 and Supplementary Table S14. In the multivariate MR analysis, HDL-C (OR = 1.08, 95% CI: 0.927–1.254, *p* = 0.331) was not significantly associated with the risk of developing female infertility after mutual adjustment. In contrast, IVW models showed that genetically predicted LDL-C (OR: 1.12, 95% CI: 1.006–1.243, *p* = 0.042) was still causally associated with a risk of female infertility. However, we did not observe significant relationships between TG and female infertility (OR: 1.10, 95% CI: 0.970–1.247, *p* = 0.142). Similarly, there was no evidence of potential pleiotropy that could reduce instrument validity in multivariable MR (Supplementary Table S15).

## 4. Discussion

In our MR study, we explored the causal relationships between the lipid-related traits and female infertility. There were some, but not entirely consistent, conclusions using univariate and multivariate two-sample MR analysis. Our primary finding was a significant association between LDL-C and female infertility, while HDL-C was non-significant. In addition, the results of studies associating TC and TG with female infertility were inconsistent, probably due to confounding factors and the correlation of all lipid-related traits.

Xu and colleagues conducted an MR analysis using genetic association data from large GWASs on lipids and female infertility, and did not find any associations between HDL-C, LDL-C, and female infertility [29]. While this study provided some informative results, it also had limitations. On the one hand, they used data from a single database, as well as relatively lenient criteria for screening genetic tools, such as acceptable mutation probabilities. On the other hand, they analyzed the association between HDL-C, LDL-C, and female infertility without considering other potential confounding causal risk factors which may have resulted in less accurate results. Compared to this study, we provided a comprehensive evaluation of the potential effects that are currently known. Even after considering the effects of BMI and AaM, our MR analysis suggested a potential causal relationship between LDL and female infertility. In addition, by integrating the results of univariable and multivariable MR analysis, we found no association between TG and female infertility, which may be explained by the correlation between TG and HDL [30].

To our knowledge, many researchers have produced different findings about the relationships between LDL-C, HDL-C, TG, TC, and female fecundability. A prospective cohort study that recruited couples from 16 counties (2005–2009) found that increased concentrations of at least four of the five lipid components in women were associated with reduced FOR and longer time-to-pregnancy, whether female serum lipids with concentrations were modeled individually or modeled jointly from both partners [31]. Meanwhile, a cohort study on serum lipid levels in women undergoing assisted reproduction found that after adjusting for potential confounding factors, differences in women’s serum lipid levels were found to ultimately lead to differences in the rates of clinical pregnancy, live birth, and miscarriage. Specifically, women with lower levels of total cholesterol, low-density lipoprotein cholesterol, and triglycerides were associated with higher rates of pregnancy and live birth [31]. Cai and colleagues conducted a secondary analysis of a randomized controlled trial with 1000 women with PCOS, and further measured the impact of preconception LDL-C, HDL-C, and TG concentrations on pregnancy outcomes. They found that each one-unit increase in serum lipid concentration was negatively associated with reproductive outcomes. They also found that increased LDL-C was independently associated with a lower chance of ovulation, clinical pregnancy, and live birth [32]. In addition, a block-randomized trial of females aged 18 to 40 years reported that poor pre-pregnancy lipid concentrations were associated with reduced fertility in women who had a previous history of pregnancy loss. The trial found that elevated levels of TG, TC, LDL-C, and decreased levels of HDL-C heightened the risk of female infertility [31]. In our study, by using multiple databases and multiple MR analysis methods, we accounted for the correlation between lipid-related traits and found that high levels of LDL-C remained associated with an increased risk of female infertility, while the impact of TG on female infertility was no longer significant.

Lipids appear to play a role in fecundability. However, the mechanisms that explain the link between serum lipids and reproductive outcomes are not yet completely comprehended. The follicle is the functional unit of the ovary that protects and nourishes the growing oocyte. Studies have demonstrated that lipid metabolism affects follicle development, oocyte maturation, and ovarian hormone function [33]. On the one hand, elevated levels of certain circulating lipids are protective factors for folliculogenesis [34]. A synthetic analogue of follicular fluid-meiosis-activating sterol (FF-MAS) was found to be a potent agonist that promotes meiotic maturation and the development of in vitro mature mouse oocytes, and improves the quality of in vitro mature mouse oocytes [35]. On the other hand, if a woman is exposed to a high-fat environment for a long time, lipid levels in the body’s oocytes may be increased, and oocyte toxicity may be induced, which can severely interfere with the process of oocyte meiosis and ultimately affect pregnancy [36]. Cholesterol metabolism is critical to female fertility, and all proliferating cells require large amounts of cholesterol for membrane synthesis. Indeed, lipoprotein cholesterol is a substrate for ovarian steroidogenesis that plays an important role in hormone levels and in the maintenance of pregnancy in mammals [37]. The large amount of cholesterol that needs to be transported to the follicular cells and eventually to the oocyte can have an effect on female mammalian reproduction, so its deficiency and excess are both detrimental [38]. Many studies have demonstrated through mouse models that abnormal lipoprotein cholesterol metabolism reduces female fertility [39]. For example, low-density lipoprotein receptors (LDLRs) have been shown to play an important role in lipoprotein metabolism [40]. Dyslipidemia caused by the absence of LDLRs significantly reduced estrogen levels and fertility in female mice [41]. It was found that the cholesterol homeostasis of oocytes in women may also be linked to the egg’s developmental potential [42]. Furthermore, studies have shown that excessive circulating HDL-C and UC accumulation in oocytes lacking HDL receptor Scavenger Class B Type I (SR-B1) hinder egg development and lead to reproductive dysfunction in female mice. This discovery not only increases the possibility that blood lipid abnormalities may lead to infertility in human females, but also suggests that targeting lipoprotein metabolism may complement current assisted reproductive technologies [43]. Liu and colleagues came to the same conclusion that excess circulating cholesterol may be the underlying cause of impaired fertility in Scarb1-deficient female mice [36]. Cholesterol accumulation and dysfunctional HDLs circulating in mouse oocytes negatively affect the egg viability of large and developmental potential, resulting in reproductive impairment [44]. Meanwhile, the liver X receptor (LXR) is a receptor primarily associated with cholesterol homeostasis. A growing number of studies describe the physiological role of LXR in female reproduction. Studies have found that reducing LXR activity exposes oocytes to higher cholesterol concentrations, further leading to abnormal oocyte maturation and atresia, leading to the development of infertility [33]. In conclusion, classical circulating lipid abnormalities, characterized by low HDL cholesterol, are generally considered to be an independent risk factor for lower fertility in women. In our study, we found evidence that genetically predicted LDL-C was associated with a lower female infertility risk, which is consistent with the conclusions of the above studies, but no obvious correlation between HDL-C and female infertility. Further studies are required to confirm the mechanism of the relationship between circulating lipids and female infertility.

This study has several strengths. First, compared to traditional observational studies, MR design can reduce the impact of reverse causal associations and potential confounding factors. At the same time, the cost and time consumed to conduct the study are relatively low, and the results obtained can be used to assess supporting evidence [31]. Second, we utilized lipid-related trait GWAS data in MVP and GLGC, which provided a sufficiently large amount of novel data to identify robust genetic variants, thus us to obtain more reliable genetic associations. Finally, to the best of our knowledge, there have been no studies utilizing MVMR to uncover any causal link between lipid-related characteristics and female infertility.

There are several limitations to this study. First, we used funnel plot symmetry, MR-Egger regression and MR-PRESSO analysis to evaluate bias due to genetic pleiotropy. Although none of them showed significant evidence, the existence of a pleiotropic effect cannot be completely excluded, which may affect the outcome by influencing other genetic variants. Second, the results of the univariate analysis of MVP and GLGC were inconsistent, possibly due to the potential heterogeneity in the lipid genetic structure. Third, the applicability of our findings to populations of non-European ancestry needs to be further investigated. Finally, our interpretation of the findings is limited by the lack of published studies comparing lipid-related characteristics and female infertility.

## 5. Conclusions

To sum up, the genetic evidence from both univariate and multivariate MR analyses suggests that LDL cholesterol is a risk factor for infertility in women. Our findings underscore the significance of interventions aimed at regulating lipid levels to prevent infertility. However, further investigation is necessary to determine the extent to which lipid-related traits impact female infertility.

## Figures and Tables

**Table 1:** Detailed information on used studies.

Exposure/Outcome	Trait	Consortium/Cohort Study	Ethnicity	Sample Sizes/Participants	Pubmed ID or Web Source
Genetic instruments for lipid-related traits in univariable MR analysis	HDL-C	MVP/GLGC	European	210,967/188,577	PubMed ID: 30275531/PubMed ID: 24097068
	LDL-C	MVP/GLGC	European	215,196/188,577	
	TC	MVP/GLGC	European	215,551/188,577	
	TG	MVP/GLGC	European	211,491/188,577	
Genetic instruments for lipid-related traits in multivariable MR analysis	HDL-C/LDL-C/TC/TG	GLGC	European	>300,000	PubMed ID: 29083408
Genetic instruments for female infertility	Female infertility	FinnGen Consortium	European	6481 samples and 75,450 controls	(https://r6.finngen.fi/, accessed on 3 February 2023)

HDL-C, high-density lipoprotein cholesterol; LDL-C, low-density lipoprotein cholesterol; TC, total cholesterol; TG, triglyceride.

## Data Availability

GWAS datasets are accessible through the ieu open GWAS pr-oject (https://gwas.mrcieu.ac.uk/, accessed on 10 January 2023) and Global Lipids Genetics Consortium Results (http://csg.sph.umich.edu/willer/public/lipids2017/, accessed on 10 January 2023).

## Supplementary Materials

The following supporting information can be downloaded at: https://www.mdpi.com/article/10.3390/nu15143130/s1, Figure S1: Scatter plot of MR effect size for causal associations between MVP lipid traits and female infertility; Figure S2: Forest plot of MR effect size using MR-Egger and IVW methods for causal associations between MVP lipid traits and female infertility; Figure S3: Funnel plot of causal associations between MVP lipid traits and female infertility; Figure S4: Leave-one-out plot to assess if a single variant is driving the associations between MVP lipid traits and female infertility; Figure S5: Scatter plot of MR effect size for causal associations between GLGC lipid traits and female infertility; Figure S6: Forest plot of MR effect size using MR-Egger and IVW methods for causal associations between GLGC lipid traits and female infertility; Figure S7: Funnel plot of causal associations between GLGC lipid traits and female infertility; Figure S8: Leave-one-out plot to assess if a single variant is driving the associations between GLGC lipid traits and female infertility; Table S1: Lipid association statistics from MVP for the effect of HDLC on female infertility in Univariate Mendelian randomization; Table S2: Lipid association statistics from MVP for the effect of LDLC on female infertility in Univariate Mendelian randomization; Table S3: Lipid association statistics from MVP for the effect of TC on female infertility in Univariate Mendelian randomization; Table S4: Lipid association statistics from MVP for the effect of TG on female infertility in Univariate Mendelian randomization; Table S5: Lipid association statistics from GLGC for the effect of HDLC on female infertility in Univariate Mendelian randomization; Table S6: Lipid association statistics from GLGC for the effect of LDLC on female infertility in Univariate Mendelian randomization; Table S7: Lipid association statistics from GLGC for the effect of TC on female infertility in Univariate Mendelian randomization; Table S8: Lipid association statistics from GLGC for the effect of TG on female infertility in Univariate Mendelian randomization; Table S9: Summary statistics for genetic instruments used in multivariable MR analyses; Table S10: Associations of HDL, LDL, TC, TG with female infertility in Univariable Mendelian randomization analysis; Table S11: MR-PRESSO for causal effect between lipid-related traits and female infertility; Table S12: Heterogeneity analyses by Cochran’s Q of univariate Mendelian randomization; Table S13: Pleiotropy analysis of univariate Mendelian randomization; Table S14: Associations of lipid traits in multivariable MR with inverse-variance weighted method; Table S15: Pleiotropy analysis using IVW of multivariable MR.

## References

[1] World Health Organization (WHO) (2018). International Classification of Diseases.

[2] Ombelet W., Cooke I., Dyer S., Serour G., Devroey P. (2008). Infertility and the provision of infertility medical services in developing countries. Hum. Reprod. Update.

[3] Zhou Z., Zheng D., Wu H., Li R., Xu S., Kang Y., Cao Y., Chen X., Zhu Y., Xu S. (2018). Epidemiology of infertility in China: A population-based study. BJOG.

[4] Liang S., Chen Y., Wang Q., Chen H., Cui C., Xu X., Zhang Q., Zhang C. (2021). Prevalence and associated factors of infertility among 20-49 year old women in Henan Province, China. Reprod. Health.

[5] Broughton D.E., Moley K.H. (2017). Obesity and female infertility: Potential mediators of obesity’s impact. Fertil. Steril..

[6] Finelli R., Mottola F., Agarwal A. (2021). Impact of Alcohol Consumption on Male Fertility Potential: A Narrative Review. Int. J. Environ. Res. Public. Health.

[7] Wesselink A.K., Hatch E.E., Rothman K.J., Mikkelsen E.M., Aschengrau A., Wise L.A. (2019). Prospective study of cigarette smoking and fecundability. Hum. Reprod..

[8] Carson S.A., Kallen A.N. (2021). Diagnosis and Management of Infertility: A Review. JAMA.

[9] Ngai F.W., Lam W. (2021). Perception of family sense of coherence among Chinese couples with infertility. J. Clin. Nurs..

[10] Ngai F.W., Loke A.Y. (2022). Relationships between infertility-related stress, family sense of coherence and quality of life of couples with infertility. Hum. Fertil..

[11] Gwynne J.T., Strauss J.F. (1982). The role of lipoproteins in steroidogenesis and cholesterol metabolism in steroidogenic glands. Endocr. Rev..

[12] Bukhari S.A., Zafar K., Rajoka M., Ibrahim Z., Javed S., Sadiq R. (2016). Oxidative stress-induced DNA damage and homocysteine accumulation may beinvolved in ovarian cancer progression in both young and old patients. Turk. J. Med. Sci..

[13] Schisterman E.F., Mumford S.L., Browne R.W., Barr D.B., Chen Z., Louis G.M. (2014). Lipid concentrations and couple fecundity: The LIFE study. J. Clin. Endocrinol. Metab..

[14] Pugh S.J., Schisterman E.F., Browne R.W., Lynch A.M., Mumford S.L., Perkins N.J., Silver R., Sjaarda L., Stanford J.B., Wactawski-Wende J. (2017). Preconception maternal lipoprotein levels in relation to fecundability. Hum. Reprod..

[15] Davies N.M., Holmes M.V., Davey Smith G. (2018). Reading Mendelian randomisation studies: A guide, glossary, and checklist for clinicians. BMJ.

[16] Lin L.J., Wei Y.Y., Zhang R.Y., Chen F. (2019). Application of mendelian randomization methods in causal inference of observational study. Zhonghua Yu Fang Yi Xue Za Zhi.

[17] Pickrell J.K., Berisa T., Liu J.Z., Segurel L., Tung J.Y., Hinds D.A. (2016). Detection and interpretation of shared genetic influences on 42 human traits. Nat. Genet..

[18] Klarin D., Damrauer S.M., Cho K., Sun Y.V., Teslovich T.M., Honerlaw J., Gagnon D.R., DuVall S.L., Li J., Peloso G.M. (2018). Genetics of blood lipids among ~300,000 multi-ethnic participants of the Million Veteran Program. Nat. Genet..

[19] Willer C.J., Schmidt E.M., Sengupta S., Peloso G.M., Gustafsson S., Kanoni S., Ganna A., Chen J., Buchkovich M.L., Mora S. (2013). Discovery and refinement of loci associated with lipid levels. Nat. Genet..

[20] Hunter-Zinck H., Shi Y., Li M., Gorman B.R., Ji S.G., Sun N., Webster T., Liem A., Hsieh P., Devineni P. (2020). Genotyping Array Design and Data Quality Control in the Million Veteran Program. Am. J. Hum. Genet..

[21] Liu D.J., Peloso G.M., Yu H., Butterworth A.S., Wang X., Mahajan A., Saleheen D., Emdin C., Alam D., Alves A.C. (2017). Exome-wide association study of plasma lipids in >300,000 individuals. Nat. Genet..

[22] Burgess S., Bowden J., Fall T., Ingelsson E., Thompson S.G. (2017). Sensitivity Analyses for Robust Causal Inference from Mendelian Randomization Analyses with Multiple Genetic Variants. Epidemiology.

[23] Burgess S., Thompson S.G. (2017). Interpreting findings from Mendelian randomization using the MR-Egger method. Eur. J. Epidemiol..

[24] Bowden J., Davey Smith G., Haycock P.C., Burgess S. (2016). Consistent Estimation in Mendelian Randomization with Some Invalid Instruments Using a Weighted Median Estimator. Genet. Epidemiol..

[25] Hartwig F.P., Davey Smith G., Bowden J. (2017). Robust inference in summary data Mendelian randomization via the zero modal pleiotropy assumption. Int. J. Epidemiol..

[26] Verbanck M., Chen C.Y., Neale B., Do R. (2018). Detection of widespread horizontal pleiotropy in causal relationships inferred from Mendelian randomization between complex traits and diseases. Nat. Genet..

[27] Sanderson E., Spiller W., Bowden J. (2021). Testing and correcting for weak and pleiotropic instruments in two-sample multivariable Mendelian randomization. Stat. Med..

[28] Bowden J., Holmes M.V. (2019). Meta-analysis and Mendelian randomization: A review. Res. Synth. Methods.

[29] Xu W., You Y., Yu T., Li J. (2022). Insights into Modifiable Risk Factors of Infertility: A Mendelian Randomization Study. Nutrients.

[30] Johnson K.E., Siewert K.M., Klarin D., Damrauer S.M., Chang K.M., Tsao P.S., Assimes T.L., Maxwell K.N., Voight B.F. (2020). The relationship between circulating lipids and breast cancer risk: A Mendelian randomization study. PLoS Med..

[31] Jansen H., Lieb W., Schunkert H. (2016). Mendelian Randomization for the Identification of Causal Pathways in Atherosclerotic Vascular Disease. Cardiovasc. Drugs Ther..

[32] Cai W.Y., Luo X., Ma H.L., Shao X.G., Wu X.K. (2022). Association between preconception serum lipid concentrations and treatment outcomes in women with PCOS who underwent ovulation induction. Reprod. Biomed. Online.

[33] Dallel S., Tauveron I., Brugnon F., Baron S., Lobaccaro J.M.A., Maqdasy S. (2018). Liver X Receptors: A Possible Link between Lipid Disorders and Female Infertility. Int. J. Mol. Sci..

[34] Zarezadeh R., Nouri M., Hamdi K., Shaaker M., Mehdizadeh A., Darabi M. (2021). Fatty acids of follicular fluid phospholipids and triglycerides display distinct association with IVF outcomes. Reprod. Biomed. Online.

[35] Marín Bivens C.L., Lindenthal B., O’Brien M.J., Wigglesworth K., Blume T., Grøndahl C., Eppig J.J. (2004). A synthetic analogue of meiosis-activating sterol (FF-MAS) is a potent agonist promoting meiotic maturation and preimplantation development of mouse oocytes maturing in vitro. Hum. Reprod..

[36] Liu T., Qu J., Tian M., Yang R., Song X., Li R., Yan J., Qiao J. (2022). Lipid Metabolic Process Involved in Oocyte Maturation During Folliculogenesis. Front. Cell Dev. Biol..

[37] Stouffer R.L., Xu F., Duffy D.M. (2007). Molecular control of ovulation and luteinization in the primate follicle. Front. Biosci..

[38] Willnow T.E., Hammes A., Eaton S. (2007). Lipoproteins and their receptors in embryonic development: More than cholesterol clearance. Development.

[39] Yesilaltay A., Dokshin G.A., Busso D., Wang L., Galiani D., Chavarria T., Vasile E., Quilaqueo L., Orellana J.A., Walzer D. (2014). Excess cholesterol induces mouse egg activation and may cause female infertility. Proc. Natl. Acad. Sci. USA.

[40] Jeon H., Blacklow S.C. (2005). Structure and physiologic function of the low-density lipoprotein receptor. Annu. Rev. Biochem..

[41] Guo T., Zhang L., Cheng D., Liu T., An L., Li W.P., Zhang C. (2015). Low-density lipoprotein receptor affects the fertility of female mice. Reprod. Fertil. Dev..

[42] Fujimoto V.Y., Kane J.P., Ishida B.Y., Bloom M.S., Browne R.W. (2010). High-density lipoprotein metabolism and the human embryo. Hum. Reprod. Update.

[43] Yesilaltay A., Morales M.G., Amigo L., Zanlungo S., Rigotti A., Karackattu S.L., Donahee M.H., Kozarsky K.F., Krieger M. (2006). Effects of hepatic expression of the high-density lipoprotein receptor SR-BI on lipoprotein metabolism and female fertility. Endocrinology.

[44] Arias A., Quiroz A., Santander N., Morselli E., Busso D. (2022). Implications of High-Density Cholesterol Metabolism for Oocyte Biology and Female Fertility. Front. Cell Dev. Biol..

