# Supplementary material for: The Association between Circulating Lipids and Female Infertility Risk: A Univariable and Multivariable Mendelian Randomization Analysis
Source: Nutrients. 2023 Jul 13;15(14):3130. doi: 10.3390/nu15143130 (PMC10384410; doi:10.3390/nu15143130)

Supplementary Figure S1-S4 Legends

Figure S1. Scatter plot of MR effect size for causal associations between MVP lipid traits and female infertility. (A: HDL and female infertility; B: LDL and female infertility; C: TC and female infertility; D: TG and female infertility)

Figure S2. Forest plot of MR effect size using MR-Egger and IVW methods for causal associations between MVP lipid traits and female infertility. (A: HDL and female infertility; B: LDL and female infertility; C: TC and female infertility; D: TG and female infertility)

Figure S3. Funnel plot of causal associations between MVP lipid traits and female infertility. (A: HDL and female infertility; B: LDL and female infertility; C: TC and female infertility; D: TG and female infertility)

Figure S4. Leave-one-out plot to assess if a single variant is driving the associations between MVP lipid traits and female infertility. (A: HDL and female infertility; B: LDL and female infertility; C: TC and female infertility; D: TG and female infertility)

Figure S1. Scatter plot of MR effect size for causal associations between MVP lipid traits and female infertility.

A: HDL and female infertility

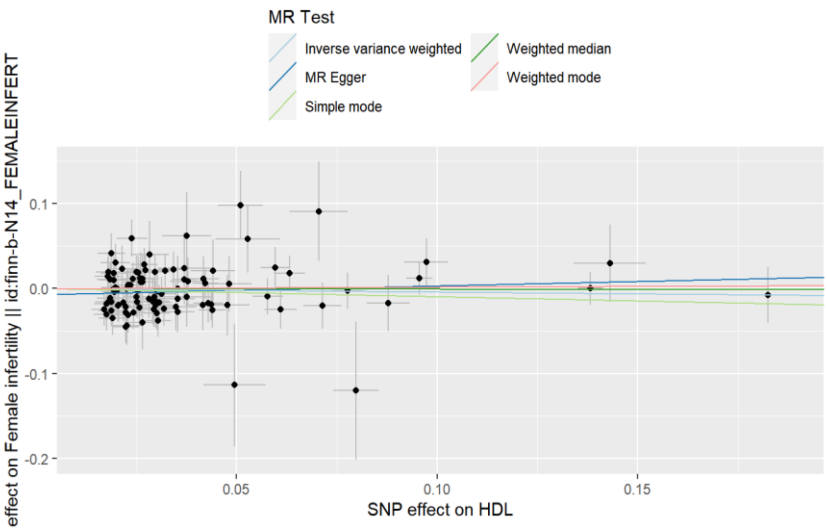

B: LDL and female infertility

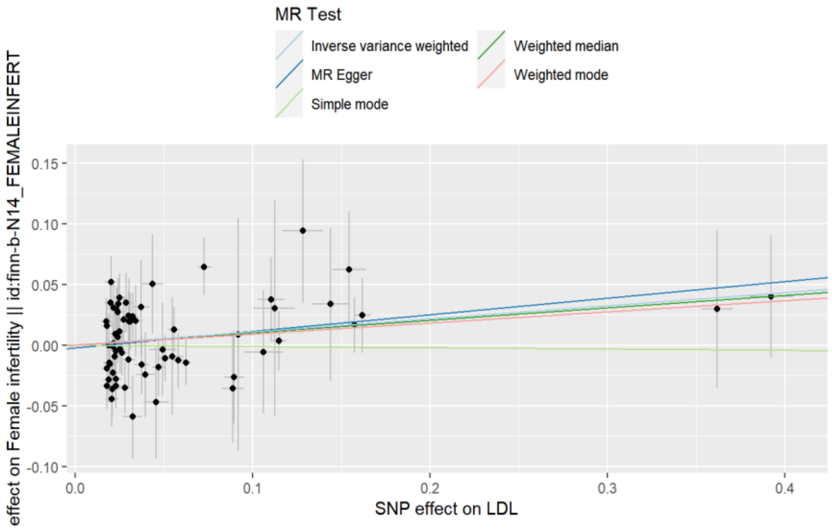

C: TC and female infertility

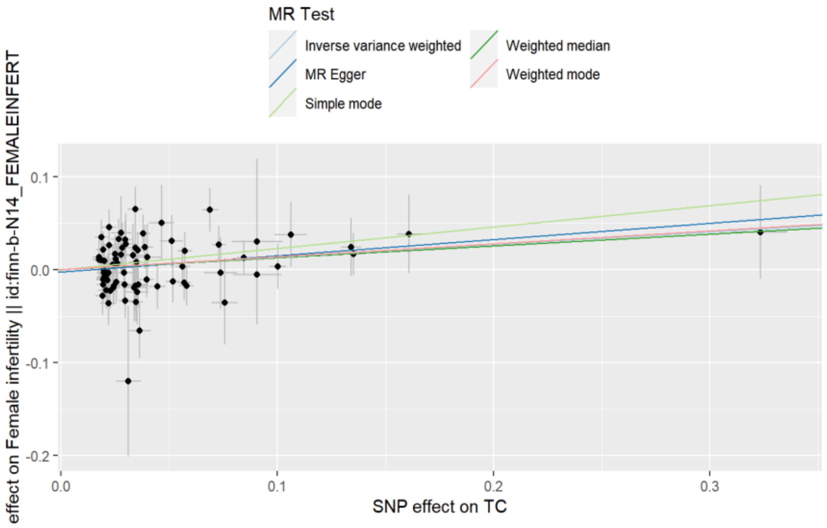

D: TG and female infertility

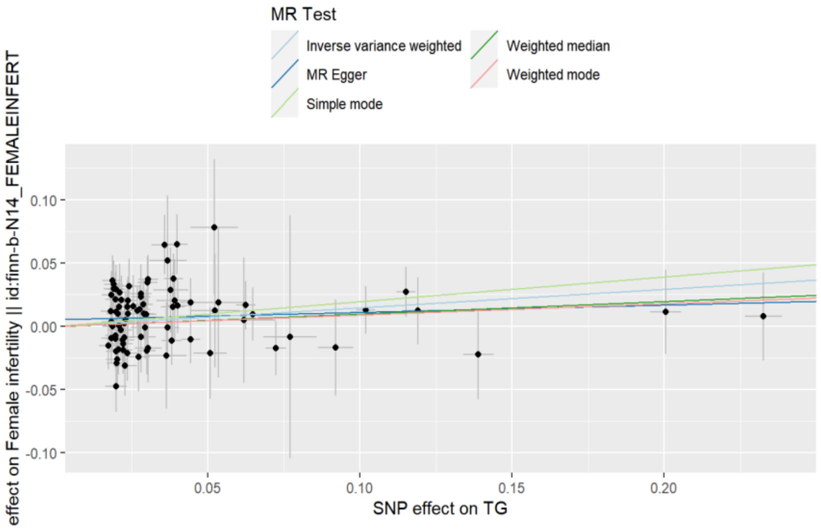

Figure S2. Forest plot of MR effect size using MR-Egger and IVW methods for causal associations between MVP lipid traits and female infertility.

A: HDL and female infertility

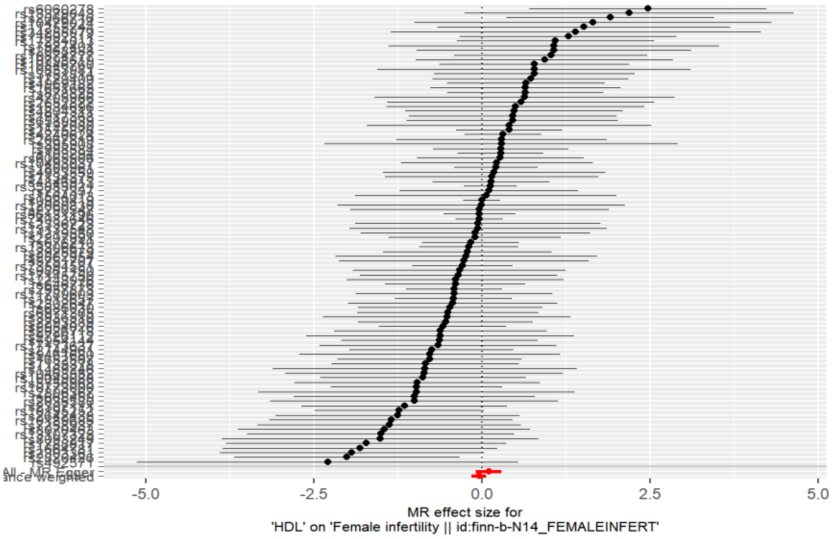

B: LDL and female infertility

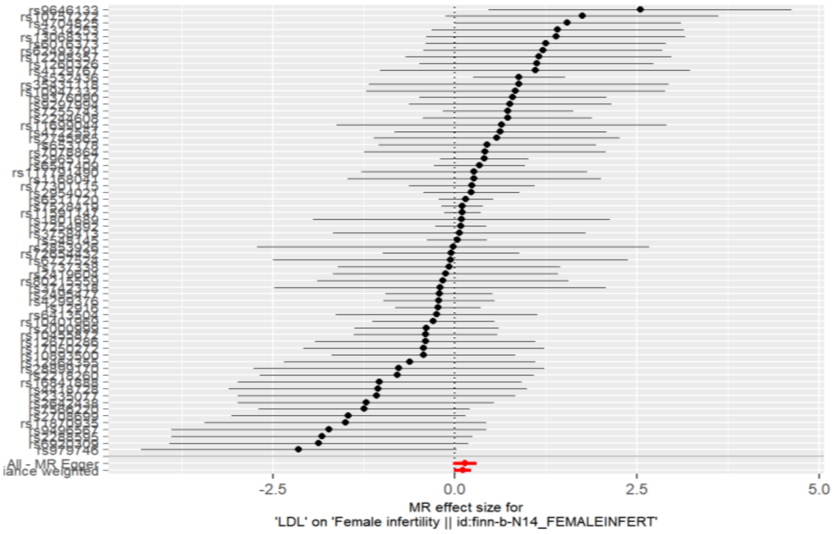

C: TC and female infertility

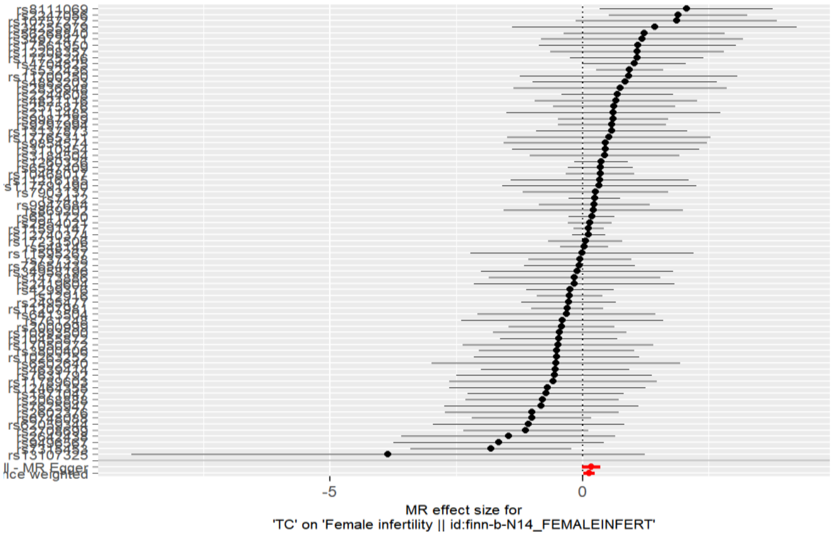

D: TG and female infertility

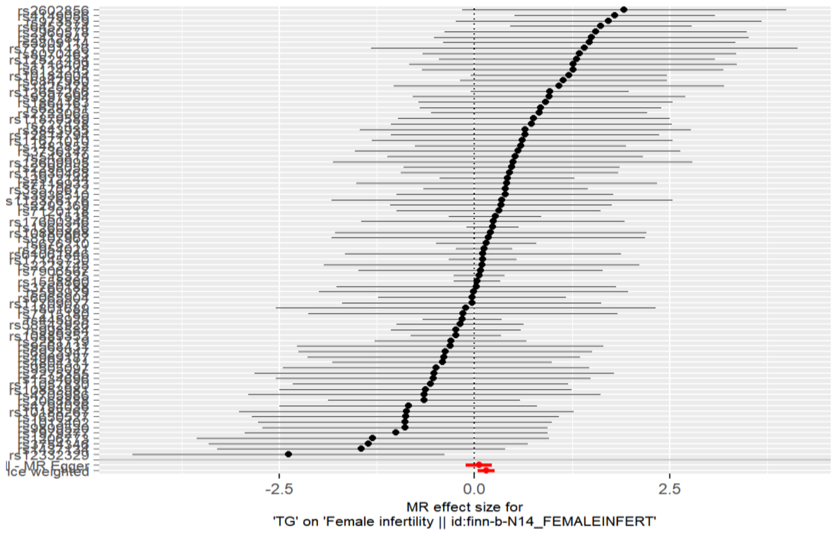

Figure S3. Funnel plot of causal associations between MVP lipid traits and female infertility.

A: HDL and female infertility

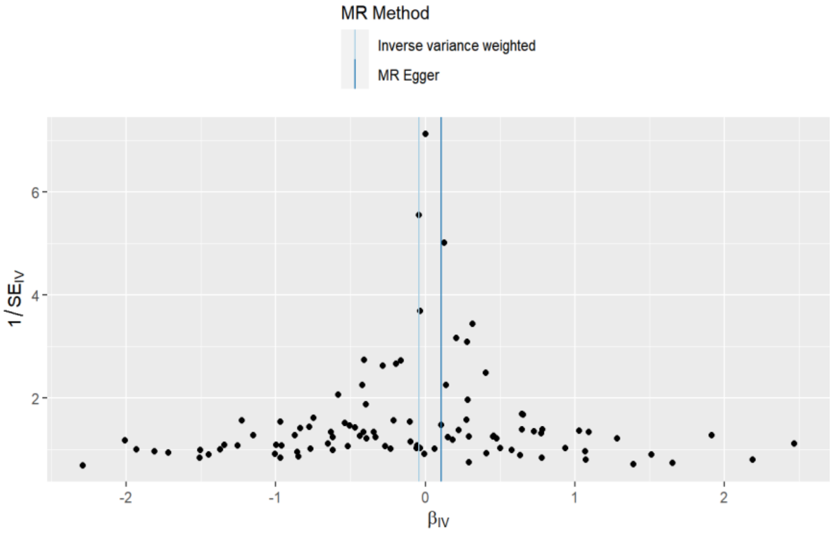

B: LDL and female infertility

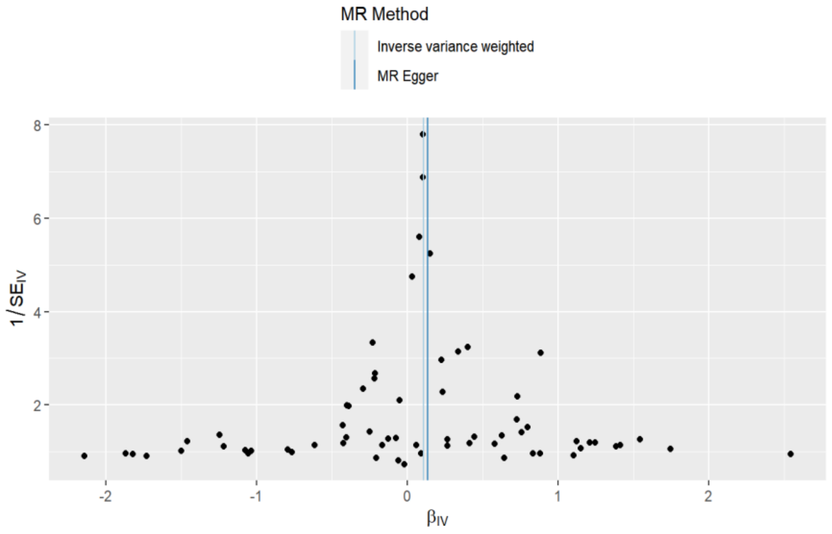

C: TC and female infertility

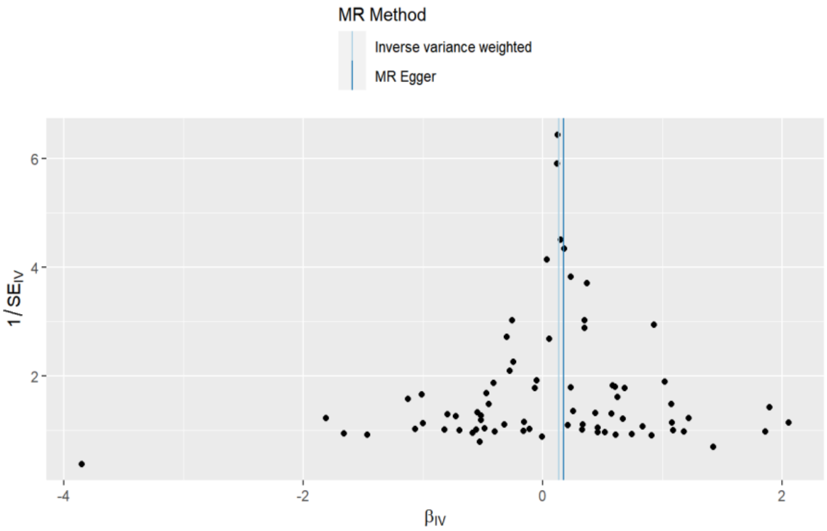

D: TG and female infertility

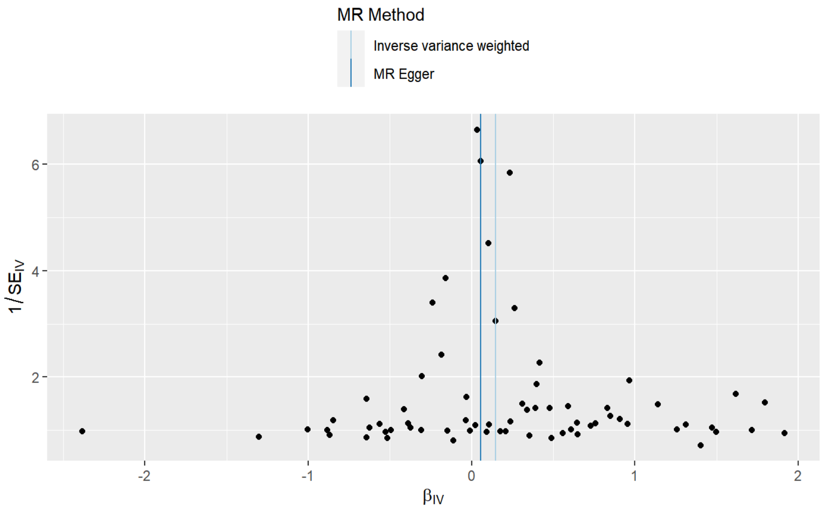

Figure S4. Leave-one-out plot to assess if a single variant is driving the associations between MVP lipid traits and female infertility.

A: HDL and female infertility

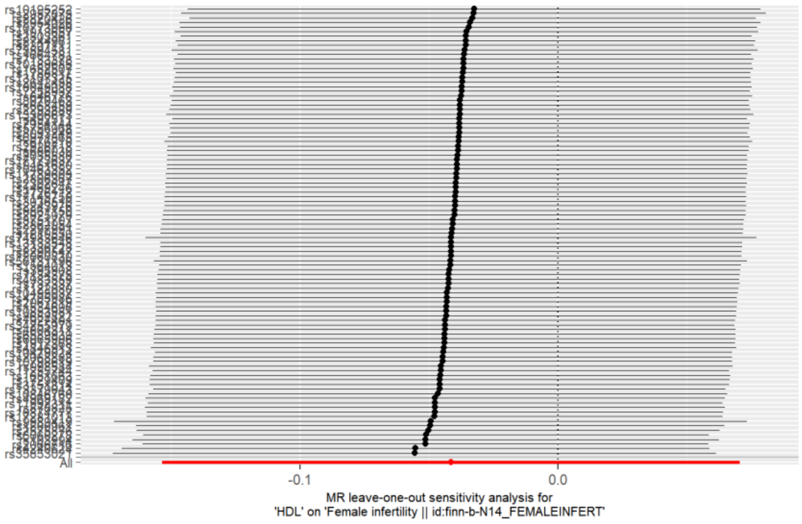

B: LDL and female infertility

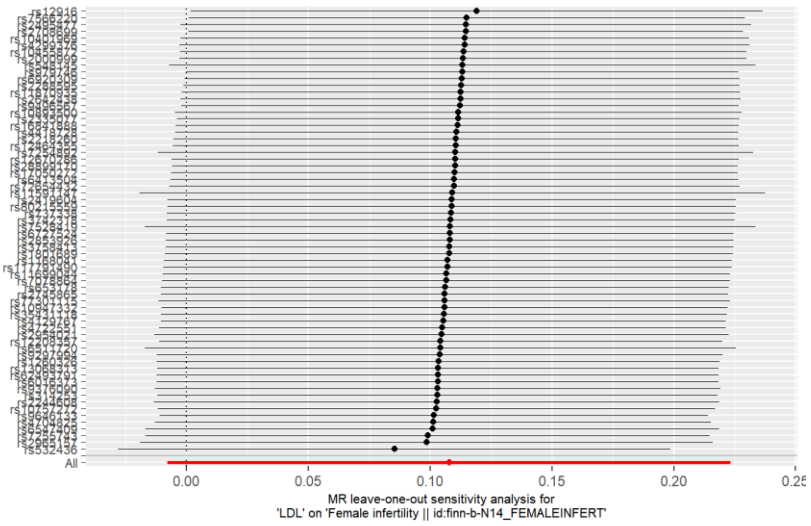

C: TC and female infertility

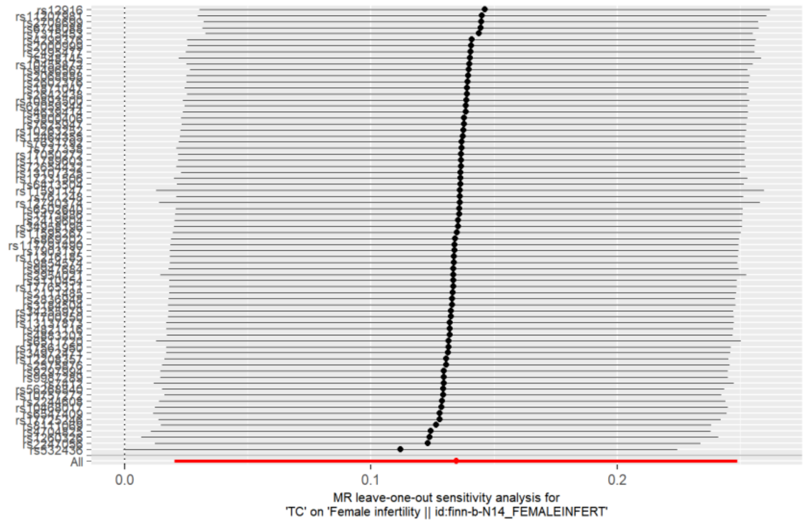

D: TG and female infertility

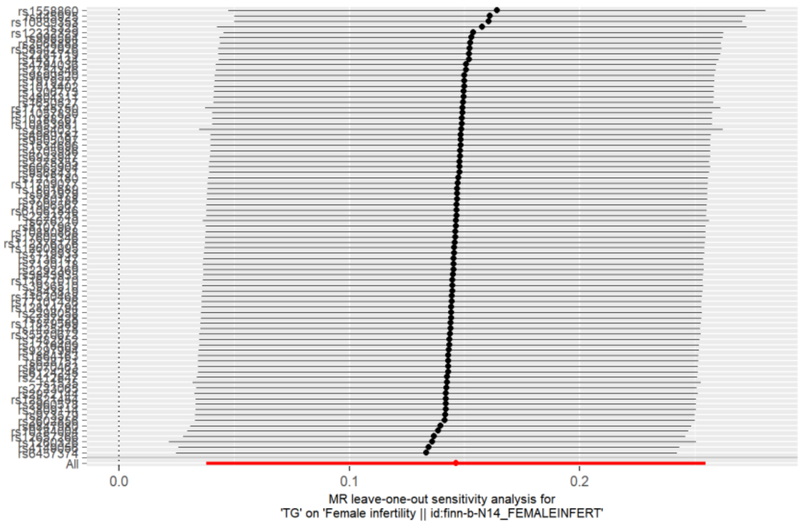

Supplementary Figure S5-S8 Legends

Figure S5. Scatter plot of MR effect size for causal associations between GLGC lipid traits and female infertility. (A: HDL-C and female infertility; B: LDL-C and female infertility; C: TC and female infertility; D: TG and female infertility)

Figure S6. Forest plot of MR effect size using MR-Egger and IVW methods for causal associations between GLGC lipid traits and female infertility. (A: HDL-C and female infertility; B: LDL-C and female infertility; C: TC and female infertility; D: TG and female infertility)

Figure S7. Funnel plot of causal associations between GLGC lipid traits and female infertility. (A: HDL-C and female infertility; B: LDL-C and female infertility; C: TC and female infertility; D: TG and female infertility)

Figure S8. Leave-one-out plot to assess if a single variant is driving the associations between GLGC lipid traits and female infertility. (A: HDL-C and female infertility; B: LDL-C and female infertility; C: TC and female infertility; D: TG and female infertility)

Figure S5. Scatter plot of MR effect size for causal associations between GLGC lipid traits and female infertility.

A: HDL-C and female infertility

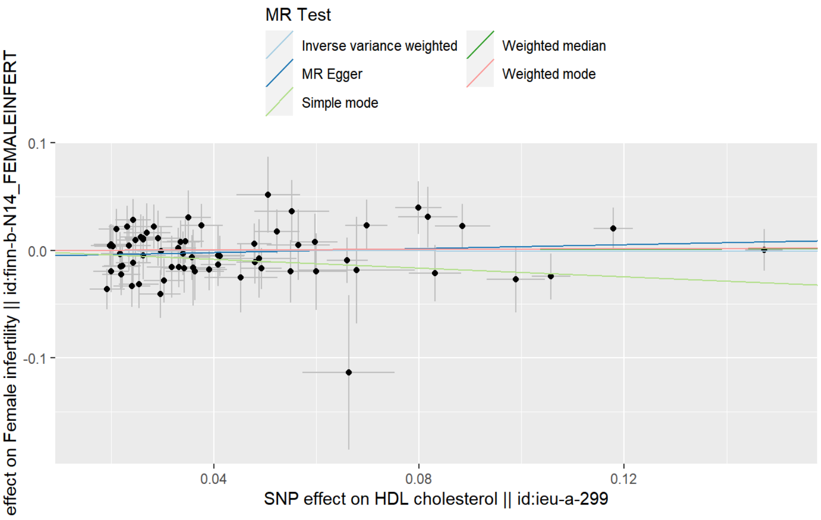

B: LDL-C and female infertility

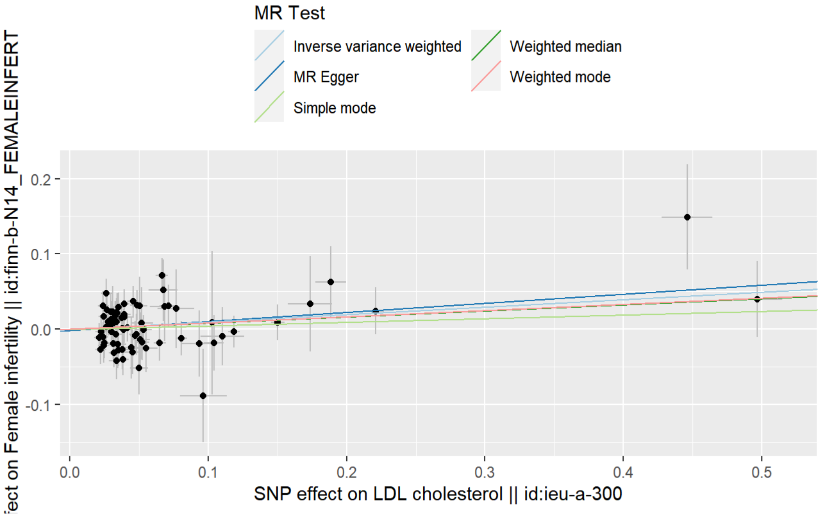

C: TC and female infertility

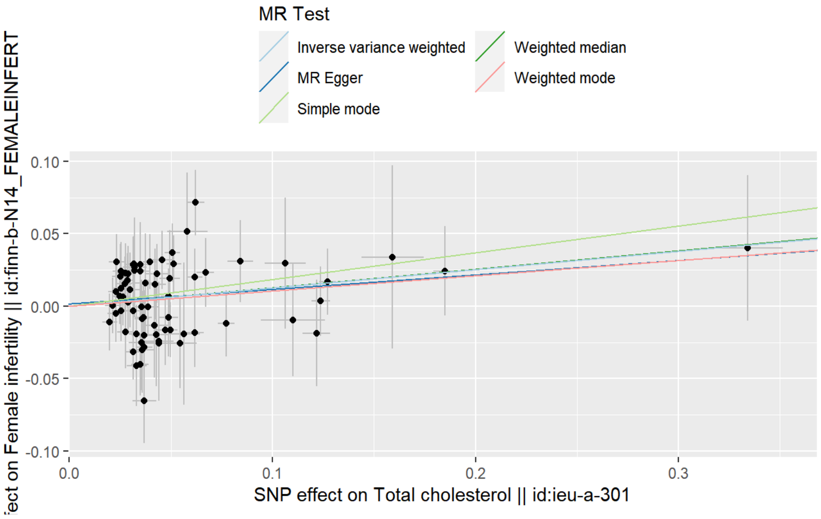

D: TG and female infertility

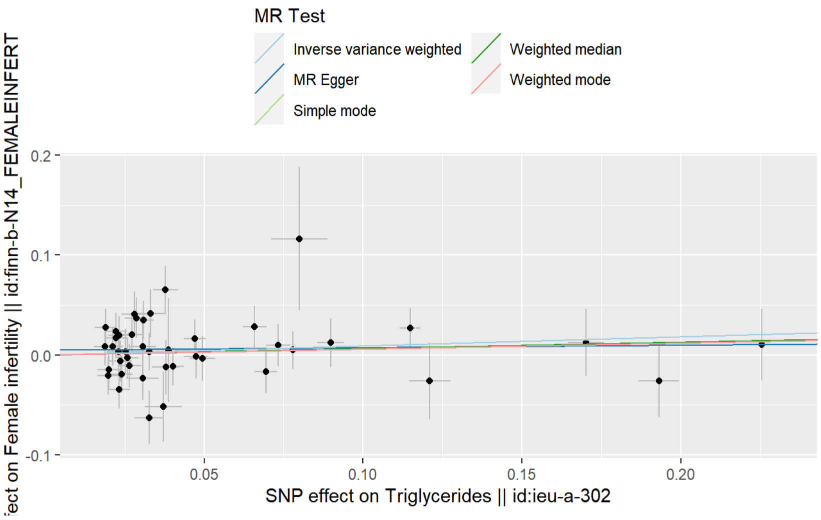

Figure S6. Forest plot of MR effect size using MR-Egger and IVW methods for causal associations between GLGC lipid traits and female infertility.

A: HDL-C and female infertility

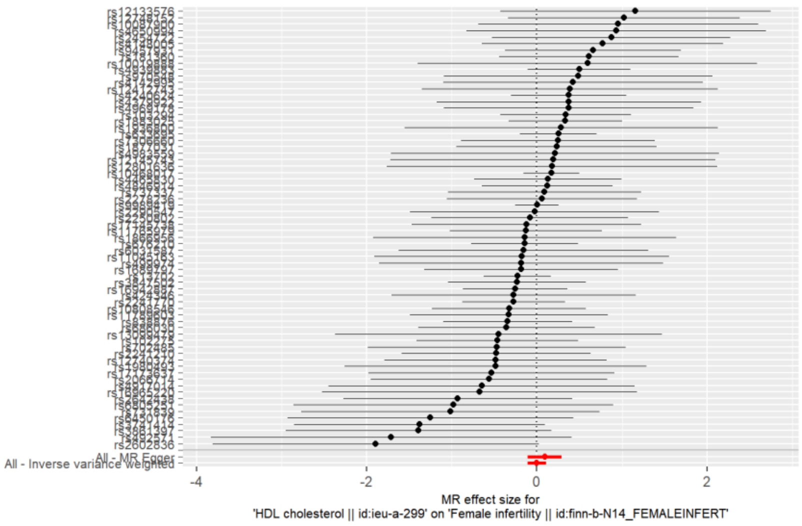

B: LDL-C and female infertility

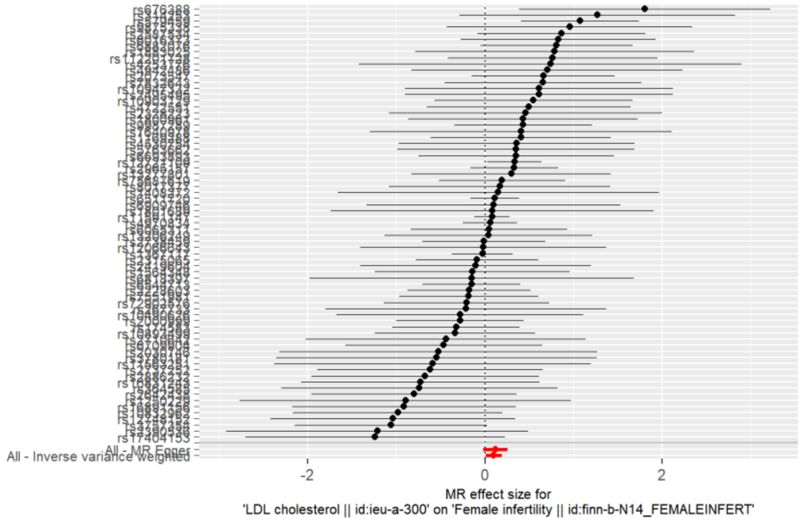

C: TC and female infertility

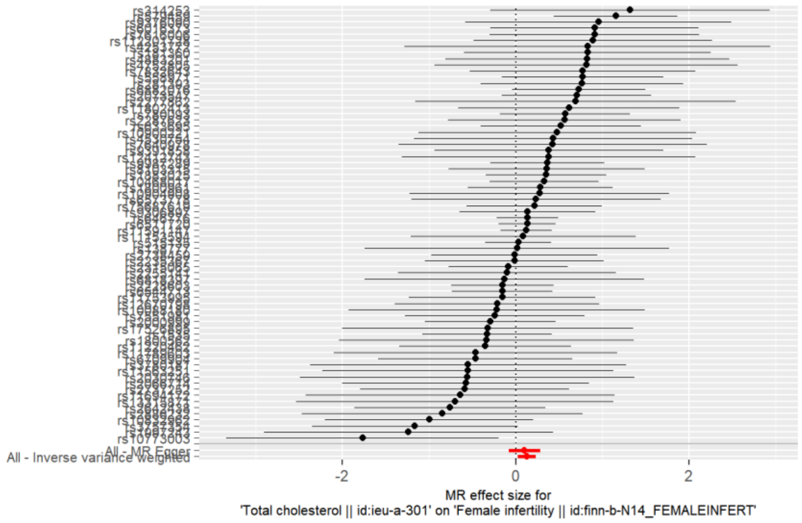

D: TG and female infertility

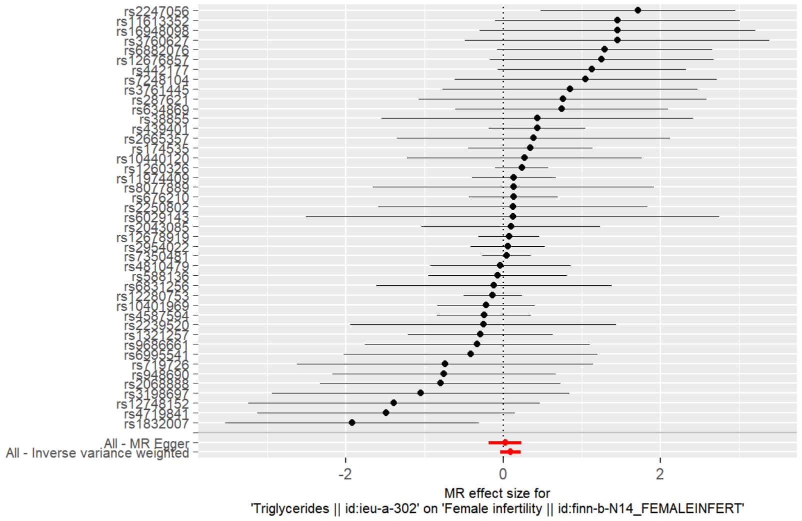

Figure S7. Funnel plot of causal associations between GLGC lipid traits and female infertility.

A: HDL-C and female infertility

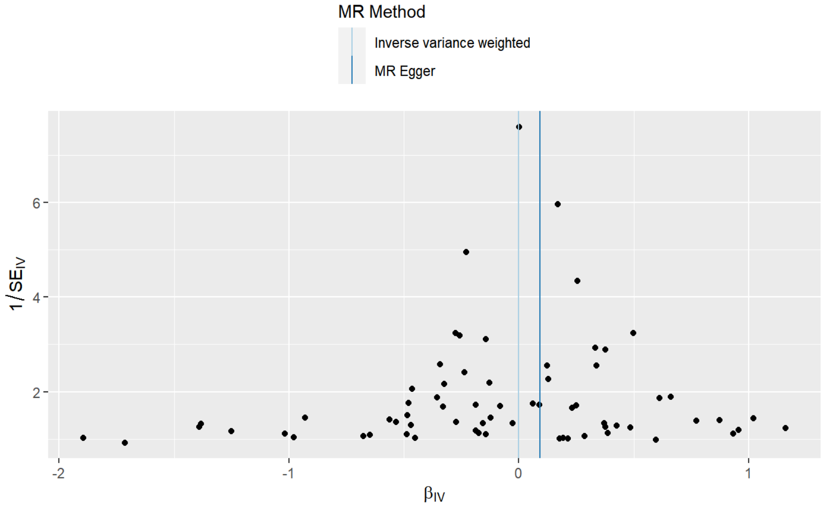

B: LDL-C and female infertility

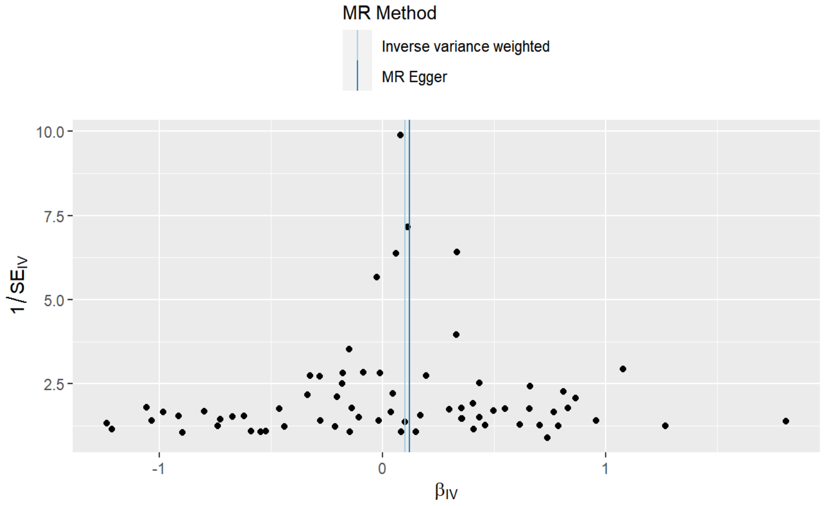

C: TC and female infertility

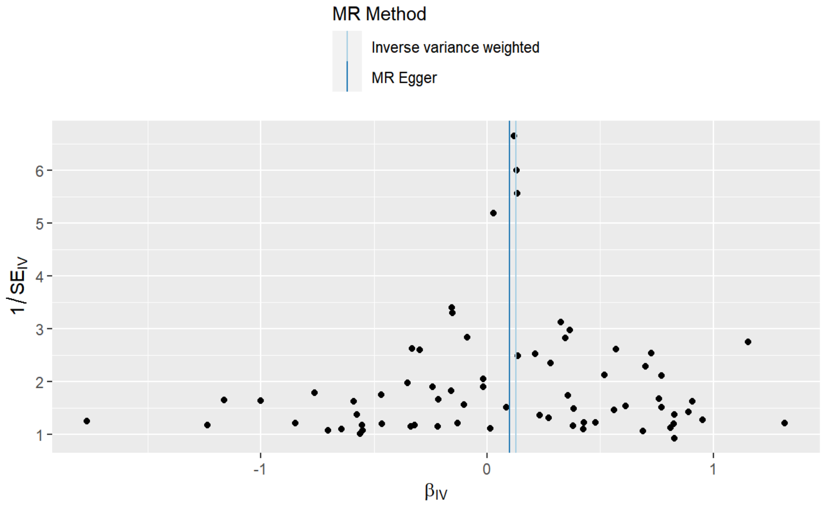

D: TG and female infertility

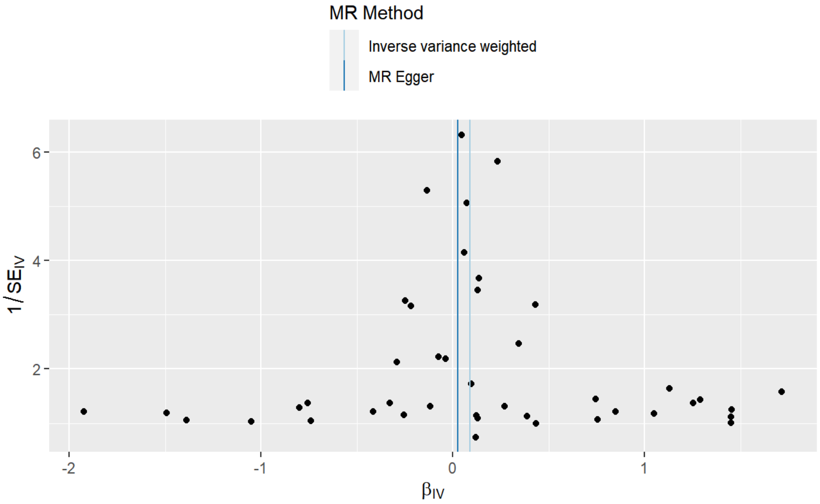

Figure S8. Leave-one-out plot to assess if a single variant is driving the associations between GLGC lipid traits and female infertility.

A: HDL-C and female infertility

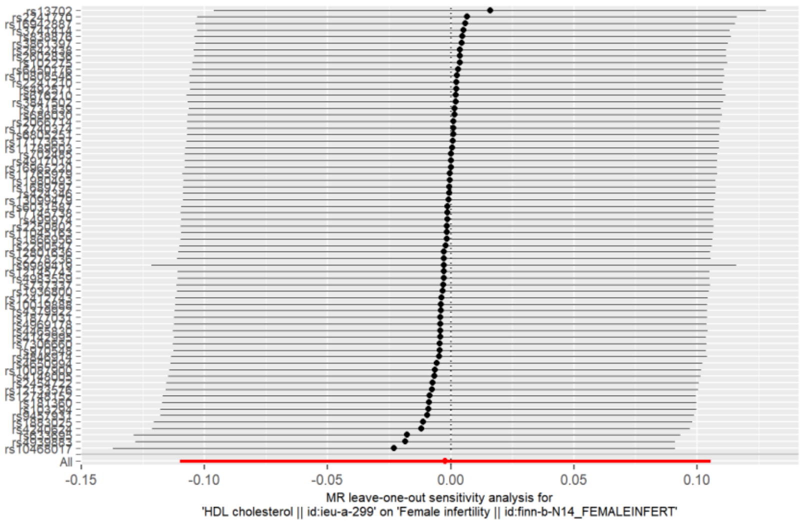

B: LDL-C and female infertility

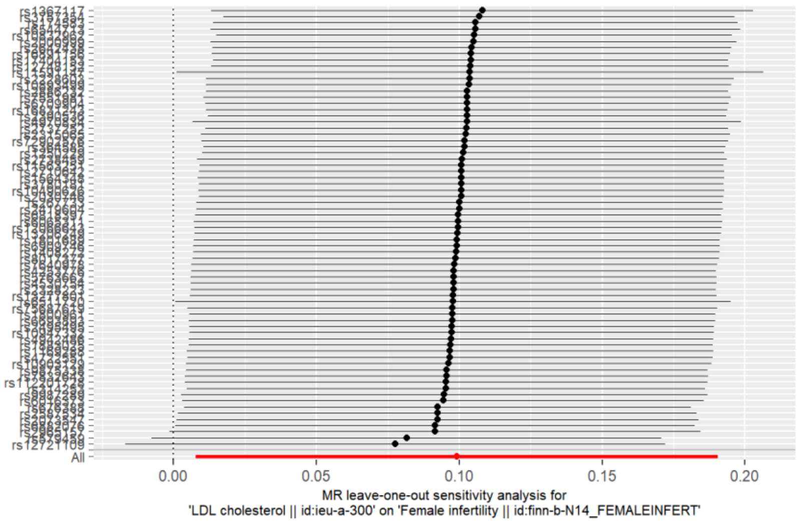

C: TC and female infertility

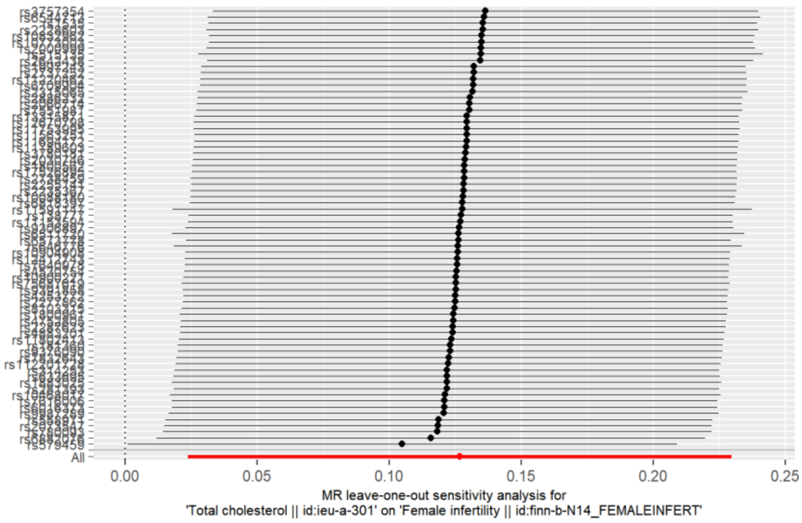

D: TG and female infertility

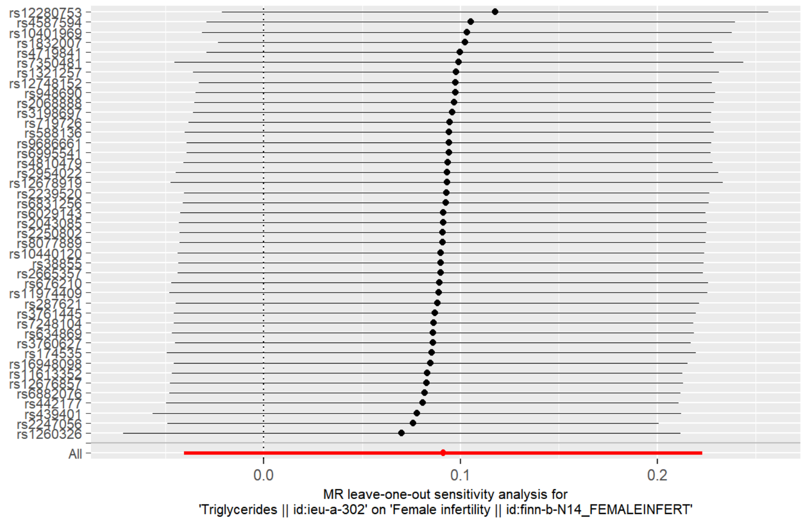

Supplement: Supplementary file 1 [file nutrients-15-03130-s001.zip › Supplementary Figures.pdf]
